# The Classification of Substance Use Disorders: Historical, Contextual, and Conceptual Considerations

**DOI:** 10.3390/bs6030018

**Published:** 2016-08-18

**Authors:** Sean M. Robinson, Bryon Adinoff

**Affiliations:** 1 Veterans Affairs North Texas Health Care System, 4500 S. Lancaster Road, Dallas, TX 75216, USA; Bryon.Adinoff@va.gov; 2 University of Texas Southwestern Medical Center, 5323 Harry Hines Blvd., Dallas, TX 75390, USA; bryon.adinoff@utsouthwestern.edu

**Keywords:** DSM, diagnostic classification, nosology, substance use disorders, historical, opioids, alcohol, cocaine, cannabis, addiction

## Abstract

This article provides an overview of the history of substance use and misuse and chronicles the long shared history humans have had with psychoactive substances, including alcohol. The practical and personal functions of substances and the prevailing views of society towards substance users are described for selected historical periods and within certain cultural contexts. This article portrays how the changing historical and cultural milieu influences the prevailing medical, moral, and legal conceptualizations of substance use as reflected both in popular opinion and the consensus of the scientific community and represented by the American Psychiatric Association’s (APA) Diagnostic and Statistical Manual of Mental Disorders (DSM). Finally, this article discusses the efforts to classify substance use disorders (SUDs) and associated psychopathology in the APA compendium. Controversies both lingering and resolved in the field are discussed, and implications for the future of SUD diagnoses are identified.

## 1. Introduction

Today, the Diagnostic and Statistical Manual of Mental Disorders (DSM) is regarded as the defining standard for mental health diagnoses (including substance use disorders (SUDs)) in America and increasingly abroad. While the fact that the DSM identifies SUDs *as primary mental health disorders* may be taken for granted today, it is noteworthy that SUDs were, prior to the third publication of the DSM (1980), largely conceptualized as manifestations of underlying primary psychopathology [1]. Thus, a large paradigm shift in SUD nosology is apparent in less than half a century’s time. Taking an even longer perspective reveals that, although psychoactive substances (including alcohol) have been around for nearly as long as recorded history, the scientific classification of SUDs only began in the early 19th century. Taken together, these observations suggest that the complex relationships human societies have had with substances over time may provide a rich and valuable backdrop of contextual and conceptual considerations for the eventual rise of nosological science. While it is beyond the scope of this article to provide a well-rounded historical account of the complex history of substance use in its entirety, the general purpose is to provide a historical framework by which the reader can contextualize and therefore better understand those influences which have shaped development of the DSM nosology of SUDs. Because the development of the DSM is singularly tied to cultural and historical developments in both Europe and the United States of America (i.e., “US”, “American”), this review takes a decidedly Western-oriented outlook on modern nosology and focuses almost exclusively on the American classification system (i.e., that which is associated with the American Psychiatric Association (APA)). Also of note: consistent with the most recent DSM, this manuscript generally uses the terminology “substance use disorder(s)” to refer to a superordinate category which is comprised of a number of singular disorders (e.g., alcohol use disorder (AUD), cannabis use disorder, etc.). In order to most effectively contrast this modern diagnostic label with earlier conceptualizations, this term is often used alongside and in comparison to earlier terms and labels.

First, a relatively brief historical overview of the long and complicated relationships humans have had with substances is provided, including the historical context of both medical and non-medical use preceding the advent of the modern diagnostic system. In order to provide a detailed yet bounded overview, this review focuses on a number of substances (including their pharmacological progenitors and/or descendants), which have, arguably, played more prominent historical roles (i.e., opium, cannabis, alcohol, cocaine). Second, this article provides demonstrative historical examples of the top-down impact that societal factors have had on substance use and substance use conceptualization and also discusses a number of influences (i.e., cultural, industrial, socio-political) which have impacted the development of the APA compendium. Third, the impact of substance use on society today is discussed in terms of substance use-related costs. Finally, following an account of the development of each version of the DSM, a few of the lingering controversies in the field are identified, and future considerations are discussed for SUD diagnoses specifically and for the addiction field as a whole.

## 2. Historical Considerations: A Long History of Psychoactive Substances

### 2.1. Opioids

**Figure 1:**
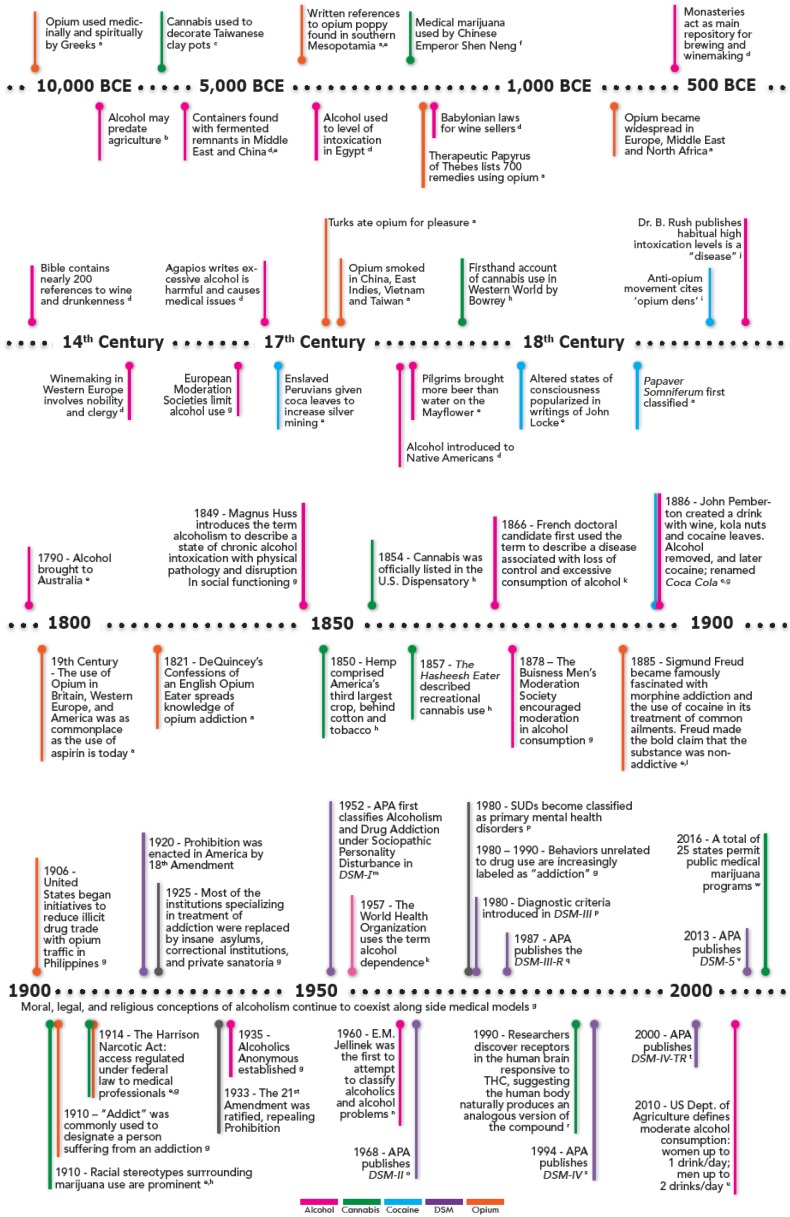
Selection of additional/supplementary historical events and perspectives related to the use of substances over time, nosological developments, and other domains relevant to the main text. Notes: ^a^ [3]; ^b^ [4]; ^c^ [5]; ^d^ [6]; ^e^ [2]; ^f^ [7]; ^g^ [8]; ^h^ [9]; ^i^ [10]; ^j^ [11]; ^k^ [12]; ^l^ [13]; ^m^ [14]; ^n^ [15]; ^o^ [16]; ^p^ [1]; ^q^ [17]; ^r^ [18]; ^s^ [19]; ^t^ [20]; ^u^ [21]; ^v^ [22]; ^w^ [23].

The history of psychoactive drugs is closely entwined with the lives and histories of the humans that cultivated and used them. Likely one of the first drugs known to humans, opium and its derivatives, have been associated with its human cultivators for millennia (for an in-depth review of the history of opium/narcotics, the reader is referred to Davenport-Hines [2] and Booth [3]). Opium itself (which contains the active opioid alkaloids *morphine* and *codeine*) is derived from a species of poppy flower, *Papaver somniferum* (Latin for “sleep inducing poppy”). Knowledge of the effects of opium in the ancient world most likely originated in Egypt, the Balkans, or the Black Sea, and the substance was obtained through relatively simple harvesting and preparation methods. The promulgation of opium throughout Persia, India, China, North Africa, and Spain by Arab traders allowed for the quick spread of the drug throughout the ancient world, leaving behind well-known written records of is properties and uses. One written account believed to reference an opium concoction, known as *nepenthe*, takes place in Homer’s *The Odyssey*, where he gives an account of “a drug that had the power of robbing grief and anger of their sting and banishing all painful memories.” This mixture may refer to opium and alcohol, a mixture later known as laudanum. Figure 1 provides additional points of historical reference. Advancing to more recent times, it was in the 19th century that experimentation with morphine for non-nonmedicinal purposes by Europeans increased while physicians concurrently came to recognize the negative effects of the drug—especially with regards to prolonged medical use.

### 2.2. Cannabis

Evidence of cannabis (a.k.a. marijuana, hemp) use dates back tens of thousands of years in both Europe and Taiwan. For detailed reviews on the history of cannabis, the reader is referred to Abel [24], Earleywine [5], Grinspoon and Bakalar [7], and Lee [9]. Although cannabis originated in Afghanistan, it was cultivated in in Europe, Arabia, France, Asia, and North and South Africa, and its use was common in the cultures comprising the modern day nations of China, Japan, India, and others. The widespread cultivation of the plant was largely due to its ability to make rope and textiles and its fibers, and was purportedly used in the creation of paper in China and Japan in the 2nd and 5th centuries A.D., respectively [25,26]. The popularity of the plant was also due to its use as a medicine; its pain relieving properties were well known in ancient China and recorded in pharmacopeias dating back to the 1st century A.D. The historical use of cannabis in the treatment of medical illness has also been documented by the ancient people of India, who used cannabis preparations to treat headaches, dysentery, and venereal disease [2]. In ancient China cannabis was used to ease the pain associated with surgery, in Japan it was used to drive away the evil spirits believed to be the cause of illness, and among the ancient Greeks it was believed to be a cure for earaches and to reduce sexual desires [24]. In 17th and 18th century Europe the use of cannabis for its purported antibiotic and analgesic effects became common and it was recognized as a sedative/hallucinogen. In India, cannabis preparations in a variety of forms were used recreationally, including *bhang*, *ganja, and* *charas* (i.e., *hashish*). As seen in Figure 1, the use of cannabis continued over time, partly as a folk remedy for a number of ailments. While its use as a recreational drug eventually became more widespread, it was not until the mid-19th century that interest in the medicinal properties of the drug once again became popular. In the US around this time, exposure to recreational use of the drug was limited to a relatively small number of individuals [5].

### 2.3. Cocaine

Although the history of cocaine itself is relatively short compared to the coca plant from which it is derived, both substances have a longstanding place in history (for reviews on the topic, the reader is referred to Davenport-Hines [2] and Karch [27]). Cocaine is one of the alkaloids contained in the leaves of the coca plant (*Erythroxylum coca*)*,* which has grown wild for thousands of years in what currently comprises the countries Colombia and Bolivia. The alkaloids were used by native peoples in modern day Peru for thousands of years to reduce hunger and thirst and to increase energy through the chewing of coca leaves. In addition to the functional utility of the leaves, they were also considered sacred by the Peruvian Incas and were used ritualistically in worship of the divine. Because the plant is cultivated under hot and humid tropical climates, it was not grown in Europe until the 1700’s, when heated greenhouses became available. In the mid-19th century, cocaine was isolated as the active ingredient in the coca leaf in Europe and cocaine was extolled by both American pharmaceutical companies as well as some notable figures in the medical community for its non-addictive qualities and its potential usefulness in weaning people off the dangerous “morphine habit” (see Figure 1 for additional selected historical events)*.* Although cocaine is famously known for being included in popular beverages in the late 19th century, the idea of cocaine as a non-addictive panacea-like wonder-drug was short-lived and since then cocaine has been employed with increasing rarity in medicine. Today, it is most commonly used by otolaryngologists as a local anesthetic with vasoconstrictive properties [28,29].

### 2.4. Alcohol

Alcohol, along with opium, is probably one of the first psychoactive substances used by man and remains one of the most widely used recreational substances. Today, the non-pathological use of alcohol today is typically associated with festivity, leisur**e**, and recreational activities, and history is replete with examples of practical and functional uses of alcohol dating back to antiquity (for detailed explications of this history see Sournia [6], Davenport-Hines [2], and White [8]). Beer and wine, for example, are believed to have been important resources for the Ancient Egyptians, with pictographs from around 4000 BCE depicting Egyptians using the substance for medicine and nutrition as well as for religious and other cultural practices. Consumption of alcohol was also pervasive throughout all segments of ancient Chinese society and its sale also provided major sources of revenue for the empire. The use of alcohol became widespread in a number of religious and cultural practices such that an imperial edict was issued stating that “moderate consumption was a religious obligation” [4]. The word *alcohol* (from the Arabic word *al-kuhl*) came to mean an essential property or spirit of something, and the mysterious properties of the substance became associated with transcendental and therefore religious experiences in a number of cultures [6,8]. The association of alcohol with religion and the divine was also common among the ancient Babylonians and the area that is now Greece. With the exception of all but a few Native American and Australian native tribes (for whom alcohol was largely non-existent prior to the arrival of Europeans), alcohol was continually consumed in large quantities throughout much of the known world. In 13th century England, as knowledge of the brewing process spread, ale became both a dietary staple for children and adults alike as well as a commodity for commerce. Alcohol use was also prominent during the renaissance and in beer was a staple of the early economy in America [2].

Along with its functional uses, alcohol was used in the ancient world, as it is today, as an intoxicant. One early account of excessive alcohol intoxication is found in the cult of Dionysus, a religious sect, which held to the idea that intoxication brought worshippers closer to their god. Indeed, consumption of alcohol, particularly wine, was so central to Greek culture that abstinence was frowned upon and wine consumption was considered a civic duty in Athens [2]. Despite the central role of alcohol in Greek society, Greeks promoted moderate drinking and reproached intoxication, with some exceptions. Similar to the Greeks, the Romans also considered wine to be central to their society and placed a high value on moderation. The decline of moderation and the rise of excessive consumption, however, began after the third century BCE, as the Roman Empire continued to spread and eventually began its period of decline [2]. As the influence of the Romans declined, so did the influence of the Christian religion rise. The growing power of the Church would exert an influence over attitudes towards drinking and intoxication for nearly two thousand years. See Figure 1 for additional selected historical events involving alcohol.

## 3. Cultural and Contextual Considerations

### 3.1. Societal Influences on Attitudes and Perceptions of Substance Use

Over time, a number of influences (i.e., religious, cultural, industrial, and sociopolitical) can be seen to impact attitudes and perceptions of substance use among both professionals and the laity alike. These attitudes and perceptions, enmeshed with the prevailing cultural zeitgeist of the time, have considerable impact across a number of domains, including interest in and funding towards treatment, the legal status/criminalization of substance users, substance use itself, as well as professional conceptualization and psychiatric nosology. A selected few of these influences are described below.

#### 3.1.1. Religious Influences

Historically speaking, the beliefs and practices of the Christian religion, for one, provided both support for the consumption of wine and also warned against excessive use [4]. Indeed, while the church held largely favorable views regarding the consumption of alcohol in moderation, it also considered over-indulgence to be a sin. According to Hanson [4],

Paul the apostle considered wine to be a creation of God and therefore inherently good and recommended its use for medicinal purposes but condemned intoxication and recommended abstinence for those who could not control their drinking.

[4] (p. 3)

The Bible itself contains nearly two thousand references to vineyards and wine, and numerous references to drinking that both condemn its use in excess and extoll its virtues in moderation [6]. As alcohol consumption remained high in colonial America, the abuse of alcohol came to be considered a sin by the church and was increasingly condemned by society [8]. The temperance movement of the late 19th century, which [30] describes as one of evangelical “moral absolutes”, left little room for consideration of moderation [30]. The movement sought to cement its cause in morality and set forth a number of arguments designed to reconcile the absolutist beliefs of the temperance movement with a number of positive references to wine in the bible (e.g., wine’s association with Jesus at the Marriage at Cana, the transfiguration of wine at communion). Although prohibition was enacted and eventually repealed, the characterological and moral problems believed to be associated with the sinful vice of excessive alcohol consumption remained. One sign, perhaps, of the perseverance of such beliefs was the groundswell of the post-prohibition grass-roots self-help group, Alcoholics Anonymous, founded in 1935 on the belief that alcoholism represented a medical disease worthy of professional attention and not societal enmity. The group simultaneously upheld the beliefs that alcoholism was both (a) a medical disease; and (b) that treatment for this disease was best accomplished through a “*moral inventory, confession of personality defects, restitution of those harmed, and the necessity of belief in and dependence upon God*” [31,32]. Today, the organization boasts more than 2 million members worldwide [33].

#### 3.1.2. Cultural Influences

As the field of mental health has come to recognize that the process of human development is inexorably linked to and fundamentally shaped by the environment in which we are enmeshed, so, too, is the ever-unfolding process of conceptualizing substance use shaped by the habits, beliefs, and traditions of the larger society. Top-down cultural influences can be seen to exert notable effects on substance use and perceptions of substance use, particularly in the 19th century. The culturally bound perception of morphine addiction of the Victorian age, for example, was enmeshed with the highly restrictive sexual attitudes towards women characteristic of the era (the same era in which psychoanalysis rose to prominence). Due to the drugs well-known effect on decreasing libido, for example, opium was often prescribed to women for the treatment of neuroses, hysteria, and hypochondriacal disorders; all of which were linked to sexual desires and frustrations among women [34,35]. Thus, the integration of societal standards regarding female sexuality into the mental health profession and diagnostic nomenclature is representative of the way in which the cultural zeitgeist at any given time can influence, if not directly promote, the misuse of substances. With the decline of the Victorian-era, cultural norms shifted, psychiatric diagnoses were re-conceptualized, and female sexuality became less restrained/is no longer treated in a ubiquitously pathological manner.

#### 3.1.3. Industrialization

The influence of industrialization upon the attitudes and perceptions of substance users is readily apparent as America progressed into the industrial revolution. The rapid change from an agricultural to an industrial economy during this time was largely a result from the establishment of the factory system, where labor was carried out by individuals in a centralized location on a large scale [36]. The already negative view of excessive consumption became magnified as society came to rely heavily upon individual personal characteristics incompatible with intoxication—namely productivity, reliability, and punctuality [4]. This was coupled with a shift in the national zeitgeist towards values consistent with the engine of the new economy, including the accumulation of materials and personal wealth. The growing antipathy surrounding the use of alcohol and substances fueled the conceptualization of the “addict” as an unproductive social outcast. Such views were only strengthened by the concomitant rise of problems typically associated with industrialization and urbanization such as increased crime, poverty, and infant mortality rates [2,4]. Furthermore, the already negative perception of “addicts” became enmeshed with moral judgment; “*Addicts were represented as self-tormenting devils lost in eternal damnation…plagued by a ‘diseased soul’*” [2] (p. 63). The near inexorable link between criminal behavior and substance use had thus been influenced by the economic concerns and industrial needs of the world’s largest burgeoning economy.

The effects of industrialization on substance use were not limited solely to alcohol, wherein excessive consumption was antagonistic to the zeitgeist of the times. Harkening back to the provision of coca leaves by the Spanish Conquistadors to the Peruvian slaves in order to increase mining of silver, the modern day equivalent of the coca leaf, cocaine, was supplied by American industrialists and plantation owners to black construction and plantation workers to increase productivity (see Figure 1). Nonetheless, the association of the drug with racial minorities resulted in racialized, zealous accounts of minorities (i.e., “*negro cocaine fiends*”) driven mad by the drug, whose use resulted in acts of murder and/or sexual depravity; not surprisingly, public disapproval of the drugs soon followed [2,37]. The propagation of such attitudes of disapproval across various strata of society would play a principal role in criminalization of substance use (including, most notably, the Temperance Movement and Prohibition). The socio-political American Temperance Movement (1817) coincided with the increasing religious and moral condemnation of alcohol use as detrimental to religious ideals and values related to family and society [4].

## 4. Legality and Morality

Recreational drug use began to be stigmatized as “socially offensive” with records referencing opium as “*the pernicious drug*” around 1814, and drug users were depicted in medical case studies and referenced as being “*incapable of self-control*” from a “*self-inflicted, self-purchased curse*” with “*no happy earthly end*” [2] (p. 62). Due to the widespread use of narcotic medications to treat wartime injuries, societies around the world found a rise in the number of addicted individuals following the American Civil War (1861–1865), the Austro-Prussian War (1866), and the Franco-Prussian War (1870–1871). Despite the growing moral intolerance of substance users, with the exception of a few US cities in the 1870’s, the possession of drugs for non-medicinal use was not a criminal offense until the early 20th century [5]. Like cocaine, cannabis became highly stigmatized in America due to its association with racial minorities and impoverished workers and, by the mid 1890’s these substances became relegated to the category of “vice” associated with criminals and the lower class. A series of laws were enacted starting in the early 20th century which criminalized the distribution of cocaine [27]. As motor vehicles became increasingly common in American early 20th century, research into the metabolic effects of alcohol on driving impairments increased, and the newfound dangers posed by alcohol intoxication took on additional costs to society [15]. As the temperance movement drew strength in industrialized America, so too did it influence attitudes abroad, with prohibition enacted in Russia (1916–1917), Hungary (1919), Norway (1919–1927), Finland (1919–1932) and the United States (1920–1933), among others [4]. Attitudes towards drug use and the increasing costs to a newly industrialized society resulted in widespread legislation designed to restrict their possession and distribution which in turn resulted in the criminalization of substance use and the entrenched association of addiction with crime, an association which has persisted (even within the mental health field). For over 30 years until its most recent iteration, the DSM has included references to legal problems as part of the criteria for SUDs (see Section 7.2).

## 5. Modern Developments

### 5.1. Opioids

In the last several decades, substantial advances in pharmacology have led to the identification of endogenous G-protein coupled opioid receptors and the use of synthetic opioids (e.g., methadone, fentanyl) and opiates (e.g., heroin, oxycodone) has proliferated, greatly increasing the amount of drugs manufactured and distributed in the United States and also abroad [38,39]. Due to their potent analgesic effect, opiate drugs have been increasingly used over the past 20 years by physicians in the treatment of chronic pain. There is a growing acceptance, however, that the long term benefits of opiates for the treatment of chronic pain are limited by analgesic tolerance, worsening of pain, the development of an opioid use disorder in those in whom the opiates were initially prescribed for chronic pain. Additionally, the diversion of prescription opioid medication is believed to have resulted in increased illicit use stemming from the subjective reduction in anxiety, mild sedation, and sense of well-being or euphoria induced by consumption of these drugs [38,39]. In 2010, about 12 million Americans (age 12 or older) reported nonmedical use of prescription painkillers during the past year, with nearly a million emergency department visits associated with prescription painkillers with an associated cost to health insurers of 72.5 billion dollars a year [40]. In 2014, over 18,000 deaths have been attributed to overdose from prescription opioid pain relievers, in addition to those associated with their illicit counterpart, heroin [41]. Today, there is increasing recognition on a national level in the U.S. of the problems associated with overuse of opioids.

### 5.2. Cannabis

Relatively recent advances in our understanding of the pharmacology of cannabis has led to the identification of its active ingredient, chemicals collectively termed *cannabinoids*, including *tetrahydrocannabinol* (THC), the chemical most associated with psychotropic effects [42]. Federal Drug Administration (FDA) approved synthetic cannabinoids are now available for the treatment of nausea/vomiting associated with chemotherapy and weight loss/loss of appetite associated with cancer and HIV/AIDS. The last several decades have also seen an unprecedented rise in physician approved marijuana use for the treatment of medical conditions in a growing number of American states [42]. Despite these advances, in 2014, it is estimated that 22.2 million Americans aged 12 or over were current users of marijuana, with 4.2 million meeting criteria for a marijuana use disorder [43].

### 5.3. Cocaine

The pharmacological properties of cocaine and related drugs are now well known and its effects on behavior are primarily attributable its effect on the neurotransmitter dopamine [28,44]. Cocaine, coca leaves, and ecgonine are presently listed as Schedule II substances by the Drug Enforcement Administration [45]. In 2014, it is estimated that 1.5 million Americans age 12 or older were current uses of cocaine (including crack cocaine), with 913,000 meeting criteria for a cocaine use disorder [43].

### 5.4. Alcohol

Alcohol is now largely used as a ritualistic and recreational intoxicant. In contrast to most illicit psychoactive substances, the health consequences of alcohol use are recognized as occurring on a continuum in which the level of potential harm is relative to the amount and pattern of an individual’s consumption. For example, while excessive use of alcohol remains the third preventable leading cause of death in the United States and contributes to over 200 diseases and health related conditions, there is also a growing recognition of the potential benefits of moderate drinking, including decreased risk of diabetes, ischemic stroke, risk heart disease and related mortality [21,46]. In 2014, slightly more than half (52.7 percent) of Americans reported current use of alcohol, with 6.4 percent of people age 12 or older having a past-year AUD [43].

## 6. Modern Classification of Substance Use Disorders: The DSM

### 6.1. DSM-I: 1952

After World War II, following the decline of German influence on psychiatric nosology, the center of psychiatry shifted to the United States and the APA commissioned its constituents to create its own psychiatric nosology [11,47]. In 1952 the first DSM (DSM-I) [14] was based upon an expanded nosology used by the United States Army created by psychoanalyst William Menninger (brother to Karl Menninger) [47,48]. Evidence of the influence of psychoanalysis and the psychosocial model in the DSM-I are evident with its observable emphasis on *psychoneurosis* and functional *reactions* to environmental stressors [11,47]. The first DSM conceptualized substance use disorder (i.e., “drug addiction” and “alcoholism”) as most commonly arising from a primary personality disorder (see Table 1) [14]. Although DSM-I conceptualized the etiology of substance use disorder as a symptom of a broader underlying disturbance, it did leave some room for exceptions—at least in coding. For example, in the case of alcoholism, the DSM did allow for a primary diagnosis of SUD when “*there is a well-established addiction to alcohol without recognizable underlying disorder*” [14]. Similarly, for drug addiction, the diagnostic label could be given “*while the individual is actually addicted*” with the “*proper personality classification to be given as an additional diagnosis*” [14]. That these exceptions were noteworthy exemptions, and not the rule, however, speaks to the strength of the etiological conceptualization of SUD as being *secondary to*, or *arising from* a primary personality disorder.

### 6.2. DSM-II: 1968

In 1959, only seven years after the publication of DSM-I, major advances in the treatment of mental disorders (i.e., the introduction of effective pharmacologic treatments) occurred in the field and, following the lead of the World Health Organization (1951), the American Medical Association (1965) recognized the severity of alcoholism and declared it to be a medical disorder. This further emphasized the need for a classification system based on the medical model [11,47,49]. The publication of the DSM-II [16] however, did little to change the influence of psychoanalysis and its characteristic descriptions of disorders described in the DSM-I. Interestingly, while the DSM-I and DSM-II did not employ diagnostic criteria as we understand them today, the DSM-II did encourage separate diagnoses for alcoholism and drug addiction “*even when it begins as a symptomatic expression of another disorder*” [40]. As seen in Table 1, three recognized types of alcoholism were recognized in DSM-II: (a) episodic excessive drinking (intoxication four times per year); (b) habitual excessive drinking (given to alcoholic persons who become intoxicated more than 12 times a year or are recognizably under the influence of alcohol more than once a week, even though not intoxicated); and (c) alcohol addiction (defined in terms of dependency, suggested by withdrawal which may be evidenced by inability to abstain for one day or heavy drinking for three months or more) [16]. Although withdrawal was emphasized for Drug Addiction, it was also recognized that dependence could occur without withdrawal (a point of semantic confusion which would follow the DSM until its most recent publication). Medically prescribed drugs were excluded in that they were taken in proportion “to the medical need” [16].

### 6.3. DSM-III: 1980

In keeping with the growing need for a valid and reliable diagnostic compendium for clinicians and researchers alike, the third edition of the DSM (DSM-III) [1] broke with psychoanalytic tradition and instituted consensus based diagnoses and diagnostic criteria [47]. These criteria, including those for SUDs, were based on the Research Diagnostic Criteria (1978) which were, in turn, influenced by the Feighner criteria (1972) [50] and earlier diagnostic attempts by Jellinek [15] to classify alcoholism. The DSM-III also saw the addition of new diagnoses (e.g., Post-traumatic Stress Disorder, Attention Deficit Disorder) and the use of consensus-based diagnoses and diagnostic criteria which, although unremarkable today, were novel concepts at the time [51]. The DSM-III is thus considered a major milestone in the field, reflecting a reemergence of the medical model and the rise of research investigators as the most prominent voices within the field [35,36].

In terms of SUDs, it is notable that the new iteration appeared devoid of the term “alcoholic” and continued the trend of separately diagnosing SUDs by now setting them apart from other mental health conditions (see Table 1). While, for the first time, this version of the DSM explicitly acknowledged differences in cultural perspectives on the acceptability of substance use, it also attempted to anchor the diagnostic criteria in terms of behavioral changes “almost all subcultures would view as extremely undesirable” [1]. Starting in DSM-III, the categories of Substance Abuse and Substance Dependence were adopted, and, although little explicit explanation is offered within the manual as to the basis for adopting this distinction, it seems that the former was equated with *pathological use* (e.g., social or occupational consequences, including legal problems which may arise from car accidents due to intoxication) and the later with *physiological dependence* (i.e., tolerance or withdrawal) [51]. While the rationale behind the DSM-III’s creation of these two categories was not described in the manual, there are a number of criticisms of this paradigm by individuals ultimately tasked with subsequent DSM revisions. Among other things, they stated that the

distinction between “abuse” and “dependence” is made entirely on the basis of evidence for the presence of physiological tolerance or withdrawal…[which leaves the current system] vulnerable to powerful, swiftly changing social forces such as the tightening of laws restricting alcohol use while driving. Thus, for example, actions of a legislature in a particular state can determine the number of residents who met DSM-III criteria for a mental disorder (i.e., alcohol abuse).

[52]

Such criticisms would form the basis for recommendations to alter these categories in the next iteration.

Interestingly, some notable irregularities existed *within* the DSM-III. For example, the manual made the explicit *additional* requirements of a pathological use criterion for Alcohol and Cannabis Dependence diagnoses in addition to the main physiological criterion; the manual also stated that data was lacking in support of the main physiological criterion necessary for a Cannabis Dependence diagnosis, i.e., “the existence and significance of tolerance with regular heavy use of cannabis are controversial” [1] (p. 176). Furthermore, while Cocaine Abuse was a recognized diagnosis, Cocaine Dependence was not included “*since only transitory withdrawal symptoms occur after cessation of or reduction in prolonged use*” [1] (p. 173).

### 6.4. DSM-III-R (1987)

While the third edition of the DSM reflected, up to this point, the most profound changes in conceptualization of psychiatric nosology since its inception, its successor, the DSM-III-R also evidenced important changes. One such change was DSM-III-R’s inclusion of criterion items formerly associated with Abuse (i.e., aspects of pathological use) in the Dependence category. By grouping (pathological) behavioral dysfunctions with physiological processes in a polythetic diagnostic set, the conceptualization of the new Dependence category stood in contrast to earlier view that physiological symptoms were both necessary and sufficient for a dependence diagnosis. The DSM-III-R goes even further in separating physiological dependence from the diagnosis of Dependence, explicitly stating that “*surgical patients [who] develop a tolerance to prescribed opioids and experience withdrawal symptoms without showing any signs of impaired control over their use of opioids*” are not considered to fall in the category of Substance Dependence [17,53].

In examining the question of how such a change came about, the reader is referred back to the conceptual validity critiques of the Abuse/Dependence diagnostic sets described in the previous section. In light of these and other conceptual validity problems, recommended revisions to the DSM-III-R included elimination of the Abuse category and incorporation of elements into a newly expanded Dependence category [52]. Such a large conceptual change, they argued, would be consistent with the influential model of a *dependence syndrome* set forth in 1976 by Edwards and Gross which described a *clinical syndrome* of alcohol dependence that was comprised of physiological dependence on one axis and pathological use/behavioral consequences on the other axis *of a singular disorder* [54]. The recommendation to expand the Dependence criteria while removing the Abuse category offers some justification for the integration of the pathological use criterion into the Dependence category and the reversal of the DSM-III stance that physiological use was, in most cases, the hallmark of the disorder. As the DSM-III-R ultimately retained the Abuse category, this re-conceptualization of the mental health disorder never fully took shape. One admitted disadvantage to the re-conceptualized single disorder model was the potential for diagnostic abandonment of individuals with lower level problems who did not meet the criterion for the would-be expanded Dependence category [52]. Although possible coding schemes were set forth to circumvent this potential problem with the removal of the Abuse diagnosis [52], some suspect the pragmatic fears of diagnostic abandonment superseded validity concerns and ultimately left the Abuse category intact while at the same time advancing the *dependence syndrome’s biaxial concept…*albeit solely within the Dependence diagnosis [55].

### 6.5. DSM-IV (1994), DSM-IV-TR (2000)

As the science of mental health continued to progress, the Abuse and Dependence categories were shown to have significant limitations, including: differences in reliability and external validity, incorrect assumptions about the relationship between abuse and dependence, and the problem of “diagnostic orphans” (individuals with symptoms for whom neither diagnosis was met) [56]. The DSM-IV attempted to clarify earlier inconsistencies regarding the distinction between physiological dependence and Substance Dependence by specifying that “Neither tolerance or withdrawal is necessary or sufficient for a diagnosis of Substance Dependence” and added specifiers “With” and “Without Physiological Dependence” [19]. The DSM-IV-TR makes a number of other relatively minor revisions to the Substance Use Disorders and highlights that, compared to Substance Dependence, “*the criteria for Substance Abuse do not include tolerance, withdrawal, or a pattern of compulsive use and instead only the harmful consequences of repeated use*” [20].

### 6.6. DSM-5: 2013 (See Also Section 7)

The fifth and most recent iteration of DSM (DSM-5) [22] represented the most dramatic modifications since DSM-III with the removal of the Abuse-Dependence paradigm and important revisions to the diagnostic criteria themselves. Most notably, DSM-5 combines Abuse and Dependence into a single unified category and measures severity on a continuous scale from mild (2–3 symptoms endorsed), moderate (4–5 symptoms endorsed) and severe (6 or more symptoms endorsed) out of 11 total symptoms (versus the previous 7) (see Table 1). The shift to a unified category measured along a dimension of severity represents a notable change from the *post-hoc* categorical *severity specifiers* in the previous version and also further cements the difference between the now defunct *DSM diagnosis of Dependence* and the medical concept of *physiological dependence*, a distinction which had been increasingly emphasized over time. As reported in Hasin*, et al.* [57], a number of empirical considerations supported this change, including psychometric studies reporting the uni-dimensionality of the biaxial abuse/dependence paradigm across a number of populations. These empirical findings suggests that, contrary to the categorization of abuse and dependence as more-or-less distinct entities with different severity levels, the criterion items actuality represent a single continuum-of-severity construct. The integration of dimensional elements of classification seen here in SUD also mirrors the call for such an approach among a number of other categorical diagnostic classifications [58,59,60].

Other noteworthy changes in the DSM-5 were the addition of the *craving* criterion, the removal of the *legal problems* criterion, and the title of the chapter, which now reads “Substance-Related and Addictive Disorders” (despite the use of the term *addiction* in the title, the text reveals that “…the word [addiction] is omitted from the official DSM-5 substance use disorder diagnostic terminology because of its uncertain definition and its potentially negative connotation” [22]). The chapter also, for the first time, includes a behavioral addiction (i.e., Gambling Disorder), suggesting that a behavioral addiction has a shared underlying neurological reward systems and a compatible symptom set with SUDs [22]. These changes (i.e., craving criterion addition, legal problems criterion elimination, and the introduction of behavioral addictions) are further discussed in the section below.

## 7. Discussion

### 7.1. Potential Practical Implications of Atheoretical Nosology

While the departure from psychoanalytic etiology and adoption of atheoretical consensus based diagnostic entities in the DSM-III is regarded as one of the greatest advances in the field over the last century, the fact that the definitive manual for the diagnosis of mental disorders provides no known etiology or pathophysiology and relies, instead, on defining a disorder by its symptoms may pose a challenge not only for the field in general but for the treatment of SUDs more specifically. One way in which atheoretical classification may prove problematic is the actual clinical usage of the diagnostic criteria themselves. While in vivo studies of clinician usage of DSM-5 for substance use disorder have yet to be carried out beyond the routine clinical practice field trials, past research comparing clinical psychiatric diagnosis versus vs. structured clinical interviews revealed significant disparities in diagnoses among providers for the same patient [61]. According to a review by First et al. [61] these and other results suggest that clinicians “*were most likely making DSM diagnoses using a method other than by evaluating each of the diagnostic criteria in sequence*” [61] (p. 842)*.* This observation raises several points of consideration. First, while there are substantial benefits in utilizing consensus-driven standardized diagnostic criteria (e.g., increased reliability and validity of diagnoses, communication between providers) these benefits are significantly curtailed if clinicians do not actually adequately employ the criteria as intended. Second, while more research is needed in order to determine precisely how clinicians are arriving at diagnoses if not utilizing full diagnostic criteria, cognitive research into the clinical reasoning of clinical psychologists suggests that experienced practitioners still rely on their own particular *causal* theoretical conceptualizations despite the *atheoretical* diagnostic criteria that have been in place for well over thirty years [62]. Thus, the same lack of universally recognized etiology that was the impetus to move beyond the early psychoanalytic influence and advance to a more valid and reliable model may run contrary to innate mechanisms for conceptualizing diagnostic entities among individual providers.

Given the necessity of the diagnostic agnosticism of the DSM and the substantial benefits this model provides to both clinicians and researchers when used correctly, the question arises as to what contributions does the particular state of psychiatric nosology today have on the field as a whole and for SUD specifically? The adoption of a consensus-based symptomological approach might represent the lack of a shared etiology among professionals. Indeed, some of the major controversies (e.g., natural recovery and the disease model, abstinence and moderation/harm reduction approaches) in the field of substance use over the last several decades have inspired protracted debate [63,64,65] and may very well epitomize this lack of etiological consensus. Today, while the DSM continues to retain its etiological neutrality, the field of substance use has undoubtedly moved in the direction of explicitly emphasizing biological and disease model conceptualizations of addictive behaviors. While advocates of a strict disease model of pathology underlying SUDs point out important achievements including improved recognition of the neurobiological process involved in addiction as well as new pharmacotherapies for treating addiction [66], this conceptualization is still, today, not without its detractors [67,68].

One potential manifestation of this lack of unified etiology or conceptually-driven nosology is the “scientist-practitioner gap”, noted in the field as a whole [69,70,71,72] and in the treatment of SUDs. Within SUD treatment, this gap is exemplified in the hesitancy among some practitioners and training programs to readily adopt and promote Evidence Based Practices (EBPs) in favor of empirically unsupported alternative approaches. Differences in support for and knowledge of the effectiveness of EBPs has been shown to be related to provider level of education, institutional culture, provider type, training, academic affiliation [73,74,75,76] and, despite the effectiveness of both psychosocial and pharmacological EBPs, research has shown that their widespread adoption has remained challenging, if not controversial, in some arenas [61]. Specifically, despite increasing availability of effective pharmacologic agents and reductions in cost associated with prescription medication for SUDs, adoption of these practices are slow [77], with *“*considerable variation in adoption across publicly funded non-profit, government-owned, privately funded non-profit, and for-profit treatment centers*”* [78] (p. 164). In addition to such macro-influences, individual provider attitudes and beliefs may be another link between conceptualization of SUDs and use of EBPs, with providers with higher responsibility-focused conceptualizations of addiction holding more negative views of the use of naltrexone in the treatment of AUD [79]. Interestingly, the use of pharmacotherapies is particularly low for SUDs (i.e., AUDs) when compared to substantially higher rates of prescribing for other comorbid mental health conditions (e.g., schizophrenia, bipolar, post-traumatic stress disorder) [80], a phenomenon which might suggest larger conceptual differences among SUD providers when compared to other mental health conditions.

### 7.2. Removal of Legal Problems Criterion

One of the significant DSM-5 changes identified above (Section 6.6) is the removal of the legal problems criterion for Substance Use Disorders. The removal of the legal problems criterion was reported to reflect the low prevalence for endorsement of this item in the general population, as well as poor fit with other criteria, and little added information based on item response theory (IRT) and differential item functioning analyses [81,82,83]. In contrast to simple summations of items endorsed by an individual in determining an outcome (i.e., level of severity), IRT is used to estimate the level of information provided by a particular item and its utility in predicting that outcome [84]. The data gathered from these models suggests that legal problems were the least associated with the overarching construct when compared to the other items and model fit was actually improved when the legal problems criterion was omitted [82]. Thus, while the removal of this criterion was accomplished through the impartiality of advanced empirical models, as described above, the significance of the departure of tradition of using the legal problems criterion as a diagnostic criterion reveals the ways in which even a purportedly *atheoretical* nosology can be influenced by specific contexts and cultural changes. This point becomes particularly salient when we consider the original contextual factors (e.g., racism, industrialization) which came together in the 19th century to make the use of certain substances illegal, thereby forming the nearly inexorable link between criminality and substance use which has persisted over time despite its questionable utility in describing SUD. The historical example of the use of opium-based drugs on women from the not-so-distant Victorian age past illustrates the powerful enmeshment of legality, medical acceptance, and cultural norms which remain so saturated the culture of the time that they remain effectively invisible. Only with the benefit of time do these cultural factors reveal themselves, and, while the example of the influence of Victorian-era cultural factors on the diagnosis and treatment of mental health in women may seem part of psychiatry’s remote past, the influence of culture on nosology can be readily witnessed even in modern times.

Although not substance related, perhaps the most salient example of social norms affecting diagnosis in recent history is the diagnostic evolution of homosexuality in the DSM which was, much like early conceptualization of SUD, considered a symptom of a real psychological illness (i.e., sociopathic personality disturbance) [85,86]. Following the advent of the LGBT rights movement in the 1960s and subsequent research into the condition, the APA eventually reversed its stance on the issue and today it is recognized that the pathology of sexual behavior (which was, in part, justified by the subjective level of disturbance it caused) is related not to an underlying pathology but rather to socially accepted norms and stigmatization. Consequently, homosexuality is no longer considered a disease or a representation of underlying personality disturbance and is conceptualized from a non-pathological viewpoint (and indeed labelled differently in order to avoid the long held stigma associated with the term *homosexual* [87]. Thus, history provides clear examples of how even an atheoretical psychiatric nosology such as the DSM is vulnerable to pathologizing behavior based on socially accepted norms- norms which only come to be revealed as reflecting large scale societal biases as they change over time though shifts in generational perspectives.

In terms of legality of substance use today, we are, perhaps, in the midst of another cultural shift; along with government’s acknowledgement of disparate racial sentencing in drug crimes, there is an increased recognition today among professionals of the dissociation between legal status of drugs with their relative dangerousness to individuals and society as well as the calls for a scientifically informed drug policy [88,89,90,91,92]. Cannabis and its derivations, for example, hold the distinction of being classified as both a Schedule I (no currently accepted medical use and lack of safety) and its active ingredient, THC, in pill-form, as Schedule II (accepted medical use and high potential for abuse) [45]. Disparities can also be seen in the legal status of alcohol use which, despite its non-illicit standing, has been recognized to provide a relatively greater level of harm to individuals and society compared to illicit drugs (i.e., heroin, crack cocaine) [90]. The removal of the legal problems criterion may be reflective of a larger cultural change of increased recognition of the somewhat arbitrary division between legal status and levels of harm of substances. The removal of the legal problems criterion underscores the larger philosophical issue of relying on a fluctuating socially-constructed criterion with arguable racial and socio-economic disparity in defining an ostensibly biological disorder in an atheoretical symptom- based diagnostic manual.

### 7.3. Removal of the Abuse/Dependence Paradigm

Another significant change to the latest iteration of the DSM identified above (Section 6.4) is the removal of the Abuse/Dependence paradigm for Substance Use Disorders, a paradigm that has been present since the adoption of the DSM-III (1980). Within this paradigm Substance Abuse has been considered a “milder” form of Substance Dependence and often construed as a prodrome. Thus, while the two categories were intended to be diagnostically distinct, they were often interpreted as being related- a conceptualization which was argued in the 1970s and resurrected, albeit in a different form, in the new millennium. In making the case for the changes to the DSM-5, empirical findings derived from modern statistical models of the dimensionality of these categories was used, which found that the criteria aligned themselves on a single dimension, a single underlying construct [83,84,93,94,95]. Thus, the issue of validity was again brought into the spotlight. Similar to the socially constructed legal criterion described in the previous section, research into the validity of the Abuse category revealed a disproportionate number of cases of Abuse being diagnosed by a criterion item (i.e., hazardous drinking) which was, itself, socially biased and mediated by political factors. One study, for example, reported that out of 1385 individuals diagnosed with current alcohol abuse, 83.6% met the criteria based solely on hazardous use, with the majority (69.3%) meeting criteria through drinking and driving alone [96]. The same study found a positive relationship between socio-economic status and DSM-IV Alcohol Abuse diagnosis, which may be explained by higher income drinkers having greater access to vehicles which, in turn, may lead to higher rates of hazardous drinking and, subsequently, Alcohol Abuse [96,97,98]. Such findings recall the recommendations described earlier [52] warning of the socially constructed and therefore problematic nature of the Abuse diagnosis.

Such findings resulted in the shift to the continuum model espoused in the DSM-5, a trend which was evident in the *severity specifiers* of previous versions (in fact, the DSM-IV and IV-TR contains a disclaimer, titled “Issues in the Use of DSM-IV: Limitations of the Categorical Approach” [19]; [20] (pp. xxii, xxxi). Although the DSM-5 has been criticized by some for retooling the longstanding dichotomy, this change may be viewed, in a larger sense, as finally addressing the conceptual validity problems underlying this distinction. For example, if Abuse was best conceptualized not a standalone mental disorder but rather as one dimension of the larger construct of the dependence syndrome as described by Edwards and Gross (1976) [54], then the amalgamation of the two diagnostic entities in the DSM-5 has increased not only the empirical but the conceptual validity of this underlying construct.

While the categorical classification of substance users in the DSM was done from a etiologically agnostic standpoint, is it plausible that, because the format is consensus (vs. theoretically) driven, and because individuals are pre-disposed to cause and effect thinking [62], the DSM will always retain elements of theory (albeit indirectly) and these will likely change as culture and thinking shift over time. As once (in) famously pointed out, symptoms of mental illness are directly tied to the social and ethical culture in which they take place [99]. While the advancement of empirical inductive reasoning which prompted the shift to the current model is a step-forward in the science of classification, it is not without its limitations; some disagreements exist about relying on mathematical models to disprove clinically entrenched concepts [55] while others have raised concerns about the validity of diagnostic thresholds (i.e., mild, moderate, severe) and the arbitrariness of diagnostic cut-offs among SUD and other diagnoses [100,101,102]. Looking forward, it remains to be seen what effect this continuum of severity conceptualization has on clinical work and reliability and validity of diagnoses.

### 7.4. Addition of Craving Criterion

Another significant change in the DSM-5 identified above (Section 6.5) is the addition of the *craving* criterion. While craving has been noted in previous versions as a *feature* of the disorder, DSM-5 marks the first use of the symptom as an *actual* criterion item. According to Hasin, Fenton, Beseler, Park and Wall [57], the inclusion of craving was supported on several fronts, including its theoretical centrality in accurately describing a clinical feature of SUD, its association with cued self-administration and relapse, its well-studied role in human and animal models of substance use, its inclusion in the ICD-10, as well as the potential for pharmacotherapeutic intervention for craving and its neural substrates. Indeed, craving is often associated with increased likelihood of relapse to alcohol use, and therefore it is thought that managing craving may improve treatment outcomes. As such, a number of pharmacologic interventions have been investigated in the last several decades which target craving reduction as a mechanism to reduce substance use including acamprosate, naltrexone, disulfiram, varenicline, lamotrigine and others [103]. To date, the results of clinical studies on reducing craving have been promising although somewhat inconsistent and await future developments (e.g., the elucidation of underlying neurobiological circuits). Current hypotheses on the neurobiology of craving (i.e., Incentive-Sensitization Theory) posit that long term substance use leads to neuroadaptations which increase the incentive salience around stimuli associated with that substance which may occur independently of the changes that mediate the subjective euphoric effects as well as withdrawal, thereby resulting in subjective experience of craving even in circumstances which highly disincentivize substance use (i.e., social, occupational, recreational impairment) [104]. As craving is then, perhaps, the only criterion which may persist following protracted abstinence, future questions may arise about how to treat and code for craving and what role craving plays in identifying remission.

### 7.5. Inclusion of “Behavioral Addictions”

Since the DSM-III-R, the field has defined addictive behaviors as relating to compulsive substance use despite adverse consequences with physiological changes often present. The inclusion of behavioral addictions as psychiatric disorders likely marks the next large paradigm shift in the field of addictions and, not surprisingly, has already garnered some debate. Although the future of behavioral addictions may lack certitude as of yet, what does seem clear, from a nosological standpoint, is the eventual expansion of the conceptualization of the broader category of addictions. This is evidenced by the chapter title “Substance-Related and Addictive Disorders” and the inclusion of a behavioral addiction in the form of Gambling Disorder and discussion of Internet Gaming Disorder as an area of future research. Gambling Disorder had previously been included in Impulse Disorders Not Elsewhere Classified since the DSM-III (originally “Pathological Gambling”). That routine ingestion of a psychopharmacologic substance is not needed in conceptualizing addictive pathology may point to the growing conceptualization of addiction as the sum of a host of neuroadaptations related to dysregulation of endogenous neurotransmitters (as well as behavioral, genetic, and pyscho-social factors) of which exogenous chemicals play a historically important but potentially diminishing part as the field progresses. Indeed, the rationale presented in the DSM-5 (i.e., that Gambling Disorder has a shared underlying neurological reward systems and some “behavioral symptoms that appear comparable to those produced by the substance use disorders” [22] appears to clearly lay the groundwork for the inclusion of other behavioral addictions. In fact, the text reports that other “excessive behavioral patterns” (i.e., internet gaming, “sex addiction”, “exercise addiction”, “shopping addiction”) are not *yet* included with the rationale cited that there has not been enough peer reviewed evidence to support diagnostic criteria “needed to identify these behaviors as mental disorders” [22]. While concern has been expressed about over-pathologizing human behavior, decreasing individual responsibility, and allowing for a deluge of un- or under-supported diagnoses to saturate and hence weaken the credibility of the field [105,106,107], future research into the neurobiological substrates of impulse-related disorders and addictions may lay a more solid framework for the behavioral addictions. Epidemiological and cultural factors of behavioral addictions will likely be an area of future research, as well as identifying behavioral and pharmacological treatment targets, creating validated and reliable measures, and measuring treatment outcomes.

## 8. Conclusions

The history of psychoactive substance use is remarkably long, dating as far back, in some cases, as the recorded history of human civilization allows. Compared to the length and complexity of human interactions with psychoactive substances over millennia, the involvement of mental health in regulating the extremes associated with over-use of psychoactive substances is a relatively recent phenomenon. The official nosology of the American mental health system, the DSM, was itself a significant advancement to the field, which lacked a unified classification system. Through its early iterations, the DSM continued to mature and shed its psychoanalytic roots in the name of the development of a unified nosology. By moving to atheoretical, consensus-based diagnostic entities, the DSM-III made a much needed and significant advancements in diagnostic reliability and validity, which supported the scientific development of the field of mental health. The observation that, despite the DSM’s agnostic approach, most providers today do not conceptualize from a strictly atheoretical standpoint suggests the possibility that the greatest advancement in psychiatric in the last century may have the unintended effect of allowing room for unscientific, idiosyncratic, or disparate etiological interpretations in a field already beleaguered by lack of consensus. Despite the atheoretical nature of the DSM clinicians retain their own conceptualizations of causal etiologies of SUDs and such lack of consensus may hinder the adoption of EBPs as the field progresses.

One of the most recent developments in the DSM-5 is the removal of the legal problems criterion, a change, which may be not only driven by empirical findings but may also represent a cultural shift away from criminalizing substance users. Philosophically, such changes may signify a coming-to-terms with the socially constructed and therefore variable nature of criminal behavior, which has long been regarded as one of the characteristic descriptors of an ostensibly biological disorder. Such a change speaks to the observation that, contrary to the popular assumption that the path of social sciences is entirely objective and linear, the iterations of the DSM reveal, in fact, a progression that is susceptible to political and social influences [11]. As Kawa and Giordano [108] state,

The evolution of the DSM illustrates that what is considered to be “medical” and “scientific” is often not an immutable standard, but rather, may be variable across time and culture, and in this way contingent upon changes in dominant schools of thought.

[108] (p. 7)

While mental health disorders have characteristically lacked clearly demarcated boundaries and have so far largely defied attempts to elucidate and categorize their exclusive etiologies, an increasing number of individuals have, over time, connected such concerns to the descriptive vs. etiological nature of psychiatric nosology and the limitations inherent in maintaining such a model [107,108].

Today, the mental health field continues to defy its atheoretical nosology by developing, for example, concrete guidelines and research funding priorities to promote cross-diagnostic advancements in the etiology of mental health disorders based on translational neuroscience. This endeavor, known as Research Domain Criteria Initiative (RDoC), is bold in its unambiguously transdiagnostic approach and was developed by the National Institute of Mental Health as a direct challenge to the diagnostically agnostic categorizational approach of the DSM [109]. While RDoC has, of yet, failed to gain significant traction in the area of SUD research, it has the potential to impact the identity of the field of mental health, including the future of diagnostic classification, research priorities, and practitioner training [110]. If more readily adopted in SUD research, RDoC may be useful in expanding existing pre-clinical, and human translational approaches and could, potentially, impact the development of a new generation of SUD pharmacotherapies [111]. While such innovations might lend much needed support to a causation-based nosological system, other advancements (e.g., in statistical modeling and classification, including latent class analysis, latent profile analysis, etc.) may provide meaningful ways of understanding and classifying groups of individuals with SUDs without the need to forgo the descriptive approach. As such advances continue to develop, questions of epidemiology and, indeed, epistemology will no doubt continue to challenge the increasingly inter-connected fields of psychology, psychiatry, and neurology. In the meantime, the continued examination and quantification of objective characterological traits (e.g., impulsivity, affect dysregulation) and their neurobiological underpinnings [112,113,114] as well as the expanding field of epigenetics [115] may continue to deconstruct the historical debate of monism vs. dualism (i.e., “the mind-body problem”) which has long beleaguered epidemiology (and therefore nosology) in mental health. 

Despite, then, the growing promise and increasing allure of a truly causation-based nosology in SUD/mental health, the realization of such an undertaking may yet prove elusive for decades to come. For now, little choice remains but to continue to refine the current classification strategy in a stepwise fashion while continuously promoting a deeper understanding and appreciation of its origins and influences.

## Figures and Tables

**Table 1:** Selected Diagnostic Considerations Comparing DSM-5 to earlier versions related to Alcohol and Substance Use Disorder(s).

Category	DSM-I	DSM-II	DSM-III	DSM-III-R	DSM-IV	DSM-5
Terminology	Alcoholism; Drug Addiction	Alcoholism; Drug Dependence	Substance Use Disorders; Substance Abuse, Substance Dependence	Psychoactive Substance Use Disorders; Substance Dependence, Substance Abuse	Substance-Related Disorders; Substance Use Disorders, Substance Dependence and Substance Abuse	Substance-Related and Addictive Disorders ^a^
Categorization	Sociopathic Personality Disturbance	Personality Disorder and Certain other Non-psychotic Mental Disorders	Classified Independently	Classified Independently	Classified Independently	Classified Independently
Role of Personality Disorders (PD) in relation to SUD	Primary. Alcoholism and drug addiction considered a “reaction” (secondary diagnosis)	Primary. Although Alcoholism is secondary, additional/separate diagnosis encouraged	Personality disturbance is listed as “Associated features” which are often present, and may be intensified by the SUD ^b^	Personality disturbance is listed as “Associated features” which are often present, and may be intensified by the SUD ^c^	Antisocial and Borderline PD are listed as “associated mental disorders” which are often co-morbid with and can complicate SUDs	SUDs are commonly seen in individuals with antisocial PDs which are associated with poorer prognosis
Main Sub-categories	Not applicable ^d^	Excessive drinking (Episodic, Habitual) Alcohol addiction	Substance Abuse, Dependence	Psychoactive Substance Abuse, Dependence	Substance Abuse, Dependence	Substance Use Disorders with Severity/Specifiers
Course Specifiers	Not specified	Not specified	Continuous ^e^, Episodic ^f^, In remission ^g^, Unspecified	Partial ^h^ and Full Remission ^i^	Early Full Remission ^j^; Early Partial Remission ^k^; Sustained Full Remission ^l^; Sustained Partial Remission ^m^; On Agonist Therapy; In a Controlled Environment	Early remission ^n^; Sustained remission ^o^; On maintenance therapy; In a controlled environment
Severity Specifiers	Not specified	Not specified	Not specified	Mild, Moderate, Severe ^p^	With, Without Physiological Dependence ^q^	Mild, Moderate, Severe ^r^
Duration	Not specified	Not specified	At least one month ^s^	At least one month ^t^	Within a 12-month period ^t^	Within a 12-month period

Note: for the purposes of space and due to the similarity of DSM-IV and DSM-IV-TR, the latter was not included in this table; ^a^ “…the word [addiction] is omitted from the official DSM-5 substance use disorder diagnostic terminology because of its uncertain definition and its potentially negative connotation” [22]; ^b^ “For example, antisocial personality traits may be accentuated by the need to obtain money to purchase illegal substances. Anxiety or depression associated with Borderline Personality Disorder may be intensified as the person uses a psychoactive substance in an unsuccessful attempt to treat his or her mood disturbance (Compton et al., 2005)” [1] (p. 171); ^c^ “For example, antisocial personality traits may be accentuated by the need to obtain money to purchase illegal substances. Anxiety or depression associated with Borderline Personality Disorder may be intensified as the person uses a psychoactive substance in an unsuccessful attempt to treat his or her mood disturbance (Compton et al., 2005)” [17] (p. 171); ^d^ Alcoholism and Drug addiction viewed as a likely manifestation of other underlying disorder(s) (e.g., PD); ^e^ “More or less regular maladaptive use for over six months” [1] (p. 166); ^f^ “A fairly circumscribed period of maladaptive use, with one or more similar periods in the past” [1] (p. 166); ^g^ “Previous maladaptive use, but not using substance at present. The differentiation of this from no longer ill and from the other course categories requires consideration of the period of time since the last period of disturbance, the total duration of the disturbance, and the need for continued evaluation or prophylactic treatment” [1] (p. 166); ^h^ “During the past six months, some use of the substance and some symptoms of dependence” [17] (p. 168); ^i^ During the past six months, either no use of the substance, or use of the substance and no symptoms of dependence” [17] (p. 168); ^j^ No criteria for Abuse or Dependence met for at least one month but less than 12 months; ^k^ One or more criteria for Abuse or Dependence met for at least one month but less than 12 months (full criteria for Dependence not met); ^l^ No criteria for Abuse or Dependence met for 12 months or longer; ^m^ Full criteria for Dependence not met for 12 months or longer however one or more criteria for Abuse and Dependence have been met; ^n^ No criteria met for at least 3 months but less than 12 months (exception made for “craving” criterion); ^o^ No criteria met 12 months or longer (exception made for “craving” criterion); ^p^ Grouped under Criteria for Severity of Psychoactive Substance Dependence. In contrast to DSM-5, objective numerical count not provided; ^q^ Applies to Dependence only; ^r^ Mild: Presence of 2–3 symptoms, Moderate: Presence of 4–5 symptom, Severe: Presence of 6 or more symptoms; ^s^ Minimum duration. Specified for Abuse only; ^t^ Abuse, Dependence.
